# *N*-(4-Hy­droxy-2-nitro­phen­yl)acetamide

**DOI:** 10.1107/S2414314622002012

**Published:** 2022-02-25

**Authors:** James E. Hines III, Curtistine J. Deere, Frank R. Fronczek, Rao M. Uppu

**Affiliations:** a Department of Environmental Toxicology, College of Agriculture, Southern University and A&M College, Baton Rouge, LA 70813, USA; b Department of Chemistry, Louisiana State University, Baton Rouge, LA 70803, USA; University of Aberdeen, Scotland

**Keywords:** crystal structure, hydrogen bonding

## Abstract

The title compound, C_8_H_8_N_2_O_4_, differs in its degree of planarity from the 3-nitro isomer and also in its hydrogen-bonding pattern. Its NH group forms an intra­molecular hydrogen bond to a nitro oxygen atom, and its OH group forms an inter­molecular hydrogen bond to an amide oxygen atom, generating [10  ] chains in the crystal.

## Abstract 2

The title compound is more planar than its 3-nitro isomer in the solid state and differs in its hydrogen-bonding pattern.

## Graphical abstract

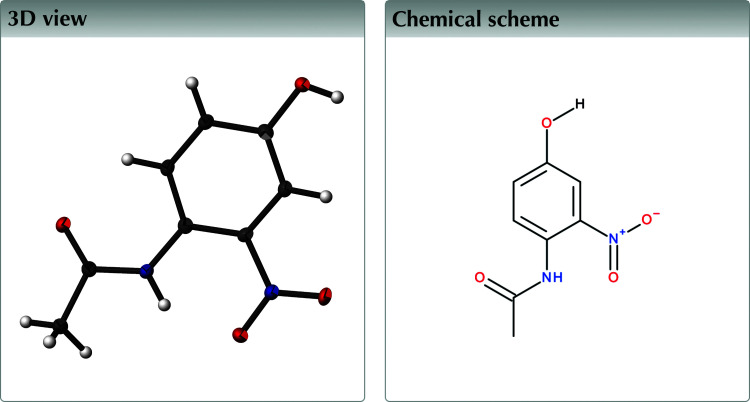

## Structure description

The putative free-radical products of the per­oxy­nitrite anion (PN)—CO_2_ reaction (^.^NO_2_ and CO_3_^.–^) have long been thought to constitute an important source of non-CYP450-mediated oxidative biotransformation of *N*-(4-hy­droxy­phen­yl)acetamide (4-HPA; acetamino­phen or paracetamol) and other xenobiotics (Babu *et al.*, 2012 ▸; Dou *et al.*, 2017 ▸; Gernapudi *et al.*, 2009 ▸; Rangan *et al.*, 2006 ▸; Uppu *et al.*, 2005 ▸). In reactions of 4-HPA/PN/CO_2_, we find that *N*-(4-hy­droxy-3-nitro­phen­yl)acetamide is one of the major products formed along with *N*,*N*′-(6,6′-dihy­droxy[1,1′-biphen­yl]-3,3′-di­yl)bis­acetamide (dimer of 4-HPA) and a metastable *N*-acetyl-1,4-benzo­quinone (NBQI; demonstrated through its binding to *N*-acetyl-l-cysteine; Uppu & Martin, 2005 ▸; Deere *et al.*, 2022 ▸). It was shown that NBQI can react with electrophiles such as the nitrite ion and form yet another nitro product, *N*-(4-hy­droxy-2-nitro­phen­yl)acetamide (Matsuno *et al.*, 1989 ▸). Although we did not find evidence for the formation of this 2-nitro isomer in 4-HPA/PN/CO_2_ reactions, we believe that this isomer along with other oxidation products of 4-HPA may play a role in the pharmacology and toxicology of 4-HPA (4-HPA overdose, either unintentional or intentional, is the most common cause of hepatic failure in the USA and elsewhere).

Towards a better understanding of this chemistry, we have synthesized *N*-(4-hy­droxy-2-nitro­phen­yl)acetamide and *N*-(4-hy­droxy-3-nitro­phen­yl)acetamide and determined their single-crystal structures. Interestingly, the 2-nitro and 3-nitro isomers have significantly different degrees of mol­ecular planarity in the solid-state and also differ in their hydrogen bonding patterns.

**Figure 1:**
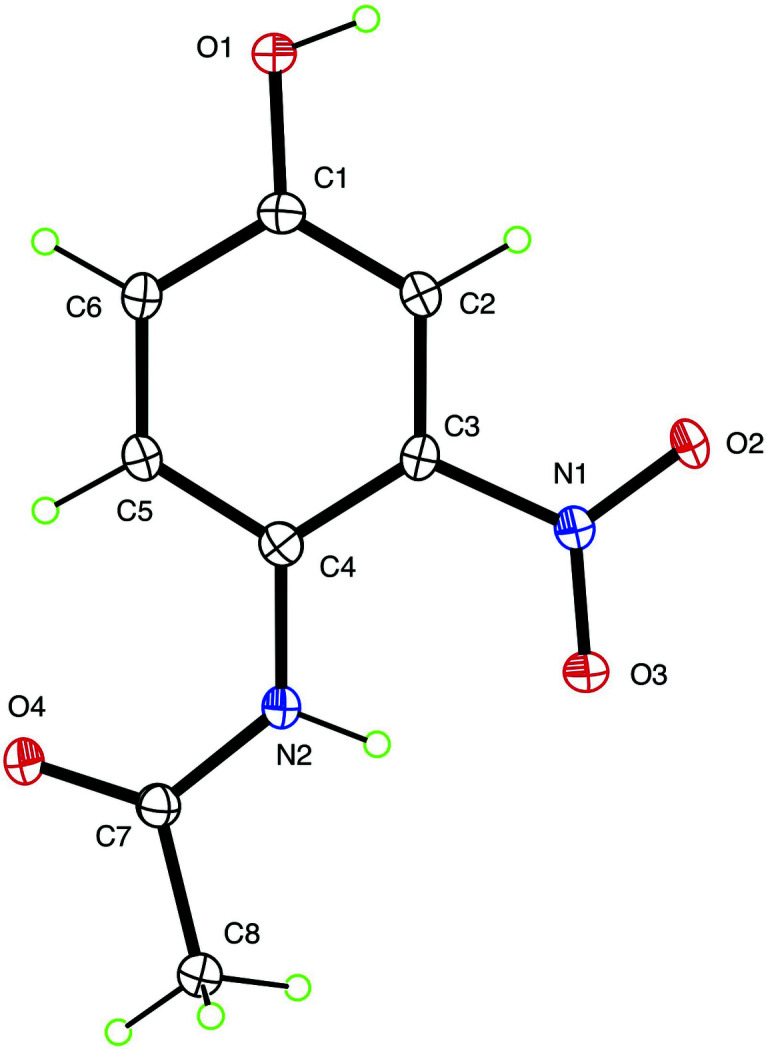
The mol­ecular structure of the title mol­ecule with 50% displacement ellipsoids.

**Figure 2:**
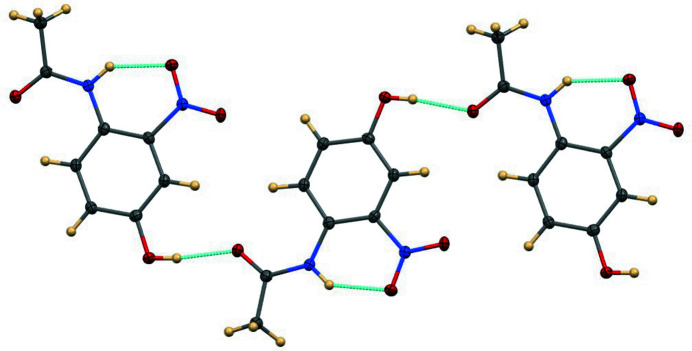
The hydrogen-bonded chain.

In *N*-(4-hy­droxy-2-nitro­phen­yl)acetamide, Fig. 1 ▸, the mol­ecule is nearly planar, with an r.m.s. deviation of 0.035 Å for the non-hydrogen atoms. The acetamido group has the largest deviation, with a 5.1 (2)° twist about its central C7—N2 bond. The N—H group forms an intra­molecular hydrogen bond (Table 1 ▸) to O3 (part of the nitro group) having an N⋯O distance of 2.6363 (15) Å and N—H⋯O angle of 139.6 (15)°. The hy­droxy group forms an inter­molecular hydrogen bond to acetamido atom O4 with O⋯O = 2.7183 (14) Å and O—H⋯O = 172.0 (18)°, thereby forming chains propagating in the [10 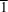 ] direction (Figs. 2 ▸ and 3 ▸).

The crystal structure of *N*-(4-hy­droxy-3-nitro­phen­yl)acetamide has been reported (Salahifar *et al.*, 2015 ▸; Deere *et al.*, 2019 ▸). It is significantly less planar than the title compound, with the acetamido group twisted out of the plane of the phenyl group by 9.0 (2)° and the nitro group twisted out of the phenyl plane by 11.8 (2)°. Its hydrogen-bonding pattern also differs, with the N—H group forming an inter­molecular hydrogen bond to the acetamido O atom [N⋯O = 2.9079 (17) Å; N—H⋯O = 176.6 (19)°]. Its OH group forms a bifurcated O—H⋯(O,O) hydrogen bond, with intra­molecular component to the adjacent nitro group [O⋯O = 2.6093 (17) Å] and a longer inter­molecular component to a nitro oxygen atom of an adjacent mol­ecule [O⋯O = 3.1421 (17) Å; Deere *et al.*, 2019 ▸].

## Synthesis and crystallization

**Figure 4:**
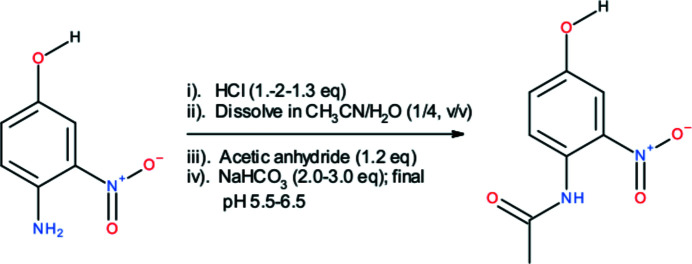
Schematic representation of the synthesis of the title compound.

The title compound was synthesized by the acetyl­ation of 4-hy­droxy-2-nitro­aniline using acetic anhydride as described by Naik *et al.* (2004 ▸) with some minor modification (Fig. 4 ▸). Briefly, 4-hy­droxy-2-nitro­aniline (3.08 g; 20 mmol) in its hydro­chloride form (prepared by addition of a slight molar excess of HCl; 26 mmol) was dissolved in 125 ml of aceto­nitrile/water (1/4, *v*/*v*). The solution was cooled in an ice bath, followed by addition of acetic anhydride (2.43 ml; 24 mmol). Then, sodium bicarbonate (3.36–5.04 g; 40–60 mmol) was added to the mixture with the contents being constantly stirred. Care was taken to maintain that the pH of the final reaction mixture was between 5.5 and 6.5. The yellow precipitate of *N*-(4-hy­droxy-2-nitro­phen­yl)acetamide was separ­ated by filtration and purified by recrystallization twice from aqueous solution. Single crystals were grown from methanol solution.

## Refinement

Crystal data, data collection and structure refinement details are summarized in Table 2 ▸.

## Supplementary Material

Crystal structure: contains datablock(s) I. DOI: 10.1107/S2414314622002012/hb4400sup1.cif

Structure factors: contains datablock(s) I. DOI: 10.1107/S2414314622002012/hb4400Isup3.hkl

Supporting information file. DOI: 10.1107/S2414314622002012/hb4400Isup3.cml

CCDC reference: 2153454

Additional supporting information:  crystallographic information; 3D view; checkCIF report

## Figures and Tables

**Figure 3:**
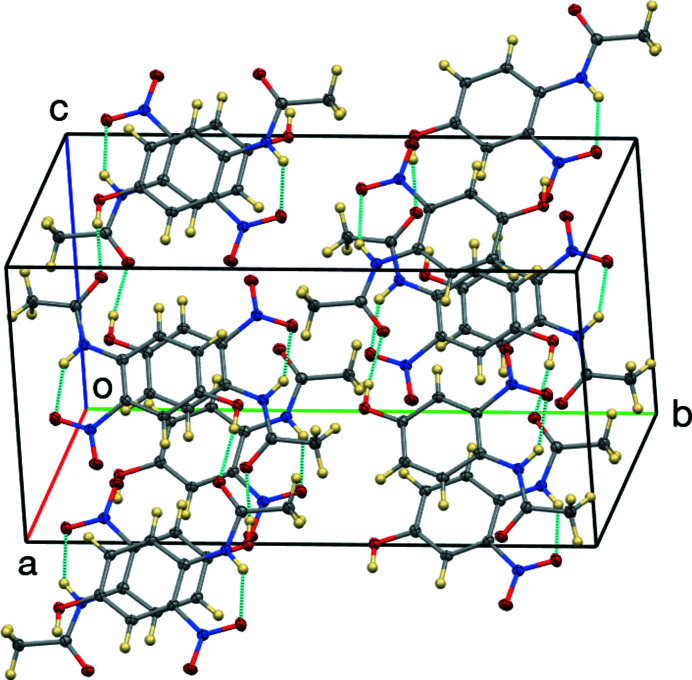
The unit cell of the title compound.

**Table 1:** Hydrogen-bond geometry (Å, °)

*D*—H⋯*A*	*D*—H	H⋯*A*	*D*⋯*A*	*D*—H⋯*A*
O1—H1*O*⋯O4^i^	0.84 (2)	1.88 (2)	2.7183 (14)	172.0 (18)
N2—H2*N*⋯O3	0.883 (19)	1.901 (17)	2.6363 (15)	139.6 (15)

Symmetry code: (i) 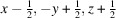 .

**Table 2:** Experimental details

Crystal data	
Chemical formula	C_8_H_8_N_2_O_4_
*M*_r_	196.16
Crystal system, space group	Monoclinic, *C*2/*c*
Temperature (K)	90
*a*, *b*, *c* (Å)	9.6643 (3), 18.5534 (5), 9.3072 (2)
β (°)	95.5075 (14)
*V* (Å^3^)	1661.13 (8)
*Z*	8
Radiation type	Cu *K*α
μ (mm^−1^)	1.10
Crystal size (mm)	0.21 × 0.07 × 0.02

Data collection	
Diffractometer	Bruker Kappa APEXII DUO CCD
Absorption correction	Multi-scan (*SADABS*; Krause *et al.*, 2015 ▸)
*T*_min_, *T*_max_	0.872, 0.978
No. of measured, independent and observed [*I* > 2σ(*I*)] reflections	6856, 1543, 1486
*R*_int_	0.028
(sin θ/λ)_max_ (Å^−1^)	0.607

Refinement	
*R*[*F* ^2^ > 2σ(*F* ^2^)], *wR*(*F* ^2^), *S*	0.035, 0.095, 1.13
No. of reflections	1543
No. of parameters	134
H-atom treatment	H atoms treated by a mixture of independent and constrained refinement
Δρ_max_, Δρ_min_ (e Å^−3^)	0.29, −0.24

Computer programs: *APEX3* and *SAINT* (Bruker, 2016 ▸), *SHELXS97* (Sheldrick, 2008 ▸), *SHELXL2014/7* (Sheldrick, 2015 ▸), *Mercury* (Macrae *et al.*, 2020 ▸); *ORTEP-3* (Farrugia, 2012 ▸) and *publCIF* (Westrip, 2010 ▸).

## full crystallographic data

### Crystal data

**Table:**

C_8_H_8_N_2_O_4_	*F*(000) = 816
*M**_r_* = 196.16	*D*_x_ = 1.569 Mg m^−^^3^
Monoclinic, *C*2/*c*	Cu *K*α radiation, λ = 1.54184 Å
*a* = 9.6643 (3) Å	Cell parameters from 5235 reflections
*b* = 18.5534 (5) Å	θ = 4.8–69.3°
*c* = 9.3072 (2) Å	µ = 1.10 mm^−^^1^
β = 95.5075 (14)°	*T* = 90 K
*V* = 1661.13 (8) Å^3^	Lath, yellow
*Z* = 8	0.21 × 0.07 × 0.02 mm

### Data collection

**Table:**

Bruker Kappa APEXII DUO CCD diffractometer	1543 independent reflections
Radiation source: IµS microfocus	1486 reflections with *I* > 2σ(*I*)
QUAZAR multilayer optics monochromator	*R*_int_ = 0.028
φ and ω scans	θ_max_ = 69.3°, θ_min_ = 4.8°
Absorption correction: multi-scan (SADABS; Krause *et al.*, 2015)	*h* = −11→7
*T*_min_ = 0.872, *T*_max_ = 0.978	*k* = −22→20
6856 measured reflections	*l* = −11→10

### Refinement

**Table:**

Refinement on *F*^2^	0 restraints
Least-squares matrix: full	Hydrogen site location: mixed
*R*[*F*^2^ > 2σ(*F*^2^)] = 0.035	H atoms treated by a mixture of independent and constrained refinement
*wR*(*F*^2^) = 0.095	*w* = 1/[σ^2^(*F*_o_^2^) + (0.0487*P*)^2^ + 1.5639*P*] where *P* = (*F*_o_^2^ + 2*F*_c_^2^)/3
*S* = 1.13	(Δ/σ)_max_ < 0.001
1543 reflections	Δρ_max_ = 0.29 e Å^−^^3^
134 parameters	Δρ_min_ = −0.23 e Å^−^^3^

### Special details

**Table:**

Geometry. All esds (except the esd in the dihedral angle between two l.s. planes) are estimated using the full covariance matrix. The cell esds are taken into account individually in the estimation of esds in distances, angles and torsion angles; correlations between esds in cell parameters are only used when they are defined by crystal symmetry. An approximate (isotropic) treatment of cell esds is used for estimating esds involving l.s. planes.
Refinement. All H atoms were located in difference maps and those on C were thereafter treated as riding in geometrically idealized positions with C—H distances 0.95 Å for phenyl and 0.98 Å for methyl. The coordinates of the N—H and O—H hydrogen atoms were refined. *U*_iso_(H) values were assigned as 1.2*U*_eq_ for the attached atom (1.5 for OH and methyl).

### Fractional atomic coordinates and isotropic or equivalent isotropic displacement parameters (Å^2^)

**Table:**

	*x*	*y*	*z*	*U*_iso_*/*U*_eq_	
O1	0.49965 (11)	0.11112 (5)	0.49610 (11)	0.0180 (2)	
H1O	0.435 (2)	0.1126 (10)	0.551 (2)	0.027*	
O2	0.36347 (11)	0.35035 (5)	0.64809 (11)	0.0240 (3)	
O3	0.47807 (11)	0.43019 (5)	0.53985 (12)	0.0239 (3)	
O4	0.79942 (10)	0.37109 (5)	0.17917 (10)	0.0208 (3)	
N1	0.44852 (12)	0.36642 (6)	0.56392 (12)	0.0163 (3)	
N2	0.65588 (12)	0.39184 (6)	0.35604 (12)	0.0149 (3)	
H2N	0.6134 (17)	0.4246 (10)	0.4050 (18)	0.018*	
C1	0.53400 (14)	0.18008 (7)	0.46502 (14)	0.0143 (3)	
C2	0.47727 (13)	0.23927 (7)	0.52723 (13)	0.0146 (3)	
H2	0.4113	0.2327	0.5955	0.018*	
C3	0.51647 (13)	0.30877 (7)	0.49012 (13)	0.0138 (3)	
C4	0.61410 (13)	0.32179 (7)	0.39015 (13)	0.0137 (3)	
C5	0.66952 (13)	0.26010 (7)	0.32904 (13)	0.0140 (3)	
H5	0.7356	0.2659	0.2606	0.017*	
C6	0.63082 (13)	0.19119 (7)	0.36548 (13)	0.0143 (3)	
H6	0.6708	0.1509	0.3220	0.017*	
C7	0.74622 (13)	0.41291 (7)	0.25993 (14)	0.0152 (3)	
C8	0.77664 (15)	0.49227 (7)	0.26014 (16)	0.0211 (3)	
H8A	0.8474	0.5036	0.3395	0.032*	
H8B	0.6914	0.5192	0.2729	0.032*	
H8C	0.8109	0.5058	0.1681	0.032*	

### Atomic displacement parameters (Å^2^)

**Table:**

	*U*^11^	*U*^22^	*U*^33^	*U*^12^	*U*^13^	*U*^23^
O1	0.0214 (5)	0.0120 (5)	0.0220 (5)	−0.0007 (4)	0.0096 (4)	0.0002 (4)
O2	0.0279 (6)	0.0189 (5)	0.0287 (6)	0.0003 (4)	0.0202 (4)	0.0005 (4)
O3	0.0298 (6)	0.0121 (5)	0.0327 (6)	−0.0014 (4)	0.0180 (5)	−0.0002 (4)
O4	0.0256 (5)	0.0158 (5)	0.0234 (5)	0.0000 (4)	0.0141 (4)	−0.0013 (4)
N1	0.0175 (6)	0.0143 (6)	0.0182 (6)	0.0003 (4)	0.0073 (4)	−0.0003 (4)
N2	0.0148 (6)	0.0129 (5)	0.0179 (6)	0.0009 (4)	0.0061 (4)	−0.0006 (4)
C1	0.0142 (6)	0.0132 (6)	0.0155 (6)	−0.0010 (5)	0.0005 (5)	0.0010 (5)
C2	0.0143 (6)	0.0159 (7)	0.0143 (6)	−0.0002 (5)	0.0041 (5)	0.0001 (5)
C3	0.0140 (6)	0.0141 (7)	0.0136 (6)	0.0017 (5)	0.0026 (5)	−0.0019 (5)
C4	0.0124 (6)	0.0153 (6)	0.0133 (6)	−0.0002 (5)	0.0004 (5)	0.0004 (5)
C5	0.0122 (6)	0.0165 (7)	0.0138 (6)	0.0003 (5)	0.0031 (5)	−0.0003 (5)
C6	0.0134 (6)	0.0152 (6)	0.0145 (6)	0.0016 (5)	0.0025 (5)	−0.0013 (5)
C7	0.0142 (6)	0.0143 (6)	0.0173 (6)	0.0006 (5)	0.0028 (5)	0.0010 (5)
C8	0.0245 (7)	0.0142 (7)	0.0266 (7)	−0.0006 (5)	0.0125 (6)	−0.0002 (5)

### Geometric parameters (Å, º)

**Table:**

O1—C1	1.3599 (16)	C2—C3	1.3966 (18)
O1—H1O	0.84 (2)	C2—H2	0.9500
O2—N1	1.2255 (15)	C3—C4	1.4081 (18)
O3—N1	1.2424 (15)	C4—C5	1.4067 (18)
O4—C7	1.2270 (17)	C5—C6	1.3835 (18)
N1—C3	1.4608 (17)	C5—H5	0.9500
N2—C7	1.3657 (18)	C6—H6	0.9500
N2—C4	1.4063 (17)	C7—C8	1.5014 (18)
N2—H2N	0.883 (19)	C8—H8A	0.9800
C1—C2	1.3795 (18)	C8—H8B	0.9800
C1—C6	1.3938 (19)	C8—H8C	0.9800
			
C1—O1—H1O	108.0 (12)	N2—C4—C3	122.20 (12)
O2—N1—O3	121.80 (11)	C5—C4—C3	115.65 (12)
O2—N1—C3	118.79 (11)	C6—C5—C4	122.03 (12)
O3—N1—C3	119.41 (11)	C6—C5—H5	119.0
C7—N2—C4	128.89 (12)	C4—C5—H5	119.0
C7—N2—H2N	119.9 (11)	C5—C6—C1	120.94 (12)
C4—N2—H2N	111.2 (11)	C5—C6—H6	119.5
O1—C1—C2	123.01 (12)	C1—C6—H6	119.5
O1—C1—C6	118.27 (12)	O4—C7—N2	123.52 (12)
C2—C1—C6	118.72 (12)	O4—C7—C8	121.83 (12)
C1—C2—C3	120.20 (12)	N2—C7—C8	114.66 (11)
C1—C2—H2	119.9	C7—C8—H8A	109.5
C3—C2—H2	119.9	C7—C8—H8B	109.5
C2—C3—C4	122.46 (12)	H8A—C8—H8B	109.5
C2—C3—N1	114.51 (11)	C7—C8—H8C	109.5
C4—C3—N1	123.02 (12)	H8A—C8—H8C	109.5
N2—C4—C5	122.14 (12)	H8B—C8—H8C	109.5
			
O1—C1—C2—C3	−179.68 (11)	N1—C3—C4—N2	1.39 (19)
C6—C1—C2—C3	0.19 (19)	C2—C3—C4—C5	0.31 (18)
C1—C2—C3—C4	−0.29 (19)	N1—C3—C4—C5	180.00 (11)
C1—C2—C3—N1	180.00 (11)	N2—C4—C5—C6	178.36 (11)
O2—N1—C3—C2	−1.17 (17)	C3—C4—C5—C6	−0.25 (18)
O3—N1—C3—C2	178.91 (11)	C4—C5—C6—C1	0.18 (19)
O2—N1—C3—C4	179.12 (12)	O1—C1—C6—C5	179.74 (11)
O3—N1—C3—C4	−0.79 (19)	C2—C1—C6—C5	−0.13 (19)
C7—N2—C4—C5	3.1 (2)	C4—N2—C7—O4	5.1 (2)
C7—N2—C4—C3	−178.38 (12)	C4—N2—C7—C8	−174.86 (12)
C2—C3—C4—N2	−178.30 (11)		

### Hydrogen-bond geometry (Å, º)

**Table:**

*D*—H···*A*	*D*—H	H···*A*	*D*···*A*	*D*—H···*A*
O1—H1*O*···O4^i^	0.84 (2)	1.88 (2)	2.7183 (14)	172.0 (18)
N2—H2*N*···O3	0.883 (19)	1.901 (17)	2.6363 (15)	139.6 (15)

Symmetry code: (i) *x*−1/2, −*y*+1/2, *z*+1/2.

## References

[bb1] Babu, S., Vellore, N. A., Kasibotla, A. V., Dwayne, H. J., Stubblefield, M. A. & Uppu, R. M. (2012). *Biochem. Biophys. Res. Commun.* **426**, 215–220. 10.1016/j.bbrc.2012.08.065 22935422

[bb2] Bruker (2016). *APEX2* and *SAINT.* Bruker AXS Inc., Madison, Wisconsin, USA.

[bb3] Deere, C. J., Hines, J. E. III, Agu, O. A. & Fronczek, F. R. (2019). *CSD Communication* (CCDC 1910293). CCDC, Cambridge, England. https://doi.org/10.5517/ccdc.csd.cc223tcd.

[bb4] Deere, C. J., Hines, J. E. III & Uppu, R. M. (2022). Unpublished.

[bb5] Dou, X., Li, J. Z., Danelisen, I., Trush, M. A., Misra, H. P., Zhu, H., Jia, Z. & Li, Y. R. (2017). *Reactive Oxygen Species* **3**, 127–134.

[bb6] Farrugia, L. J. (2012). *J. Appl. Cryst.* **45**, 849–854.

[bb7] Gernapudi, R., Babu, S., Raghavamenon, A. C. & Uppu, R. M. (2009). *FASEB J*, **23** (Suppl. 1), 397. https://faseb.onlinelibrary.wiley.com/doi/10.1096/fasebj.23.1_supplement.LB397

[bb8] Krause, L., Herbst-Irmer, R., Sheldrick, G. M. & Stalke, D. (2015). *J. Appl. Cryst.* **48**, 3–10. 10.1107/S1600576714022985 PMC4453166 26089746

[bb9] Macrae, C. F., Sovago, I., Cottrell, S. J., Galek, P. T. A., McCabe, P., Pidcock, E., Platings, M., Shields, G. P., Stevens, J. S., Towler, M. & Wood, P. A. (2020). *J. Appl. Cryst.* **53**, 226–235. 10.1107/S1600576719014092 PMC6998782 32047413

[bb10] Matsuno, T., Matsukawa, T., Sakuma, Y. & Kunieda, T. (1989). *Chem. Pharm. Bull.* **37**, 1422–1423.

[bb11] Naik, S., Bhattacharjya, G., Kavala, V. R. & Patel, B. K. (2004). *Arkivoc*, pp. 55–63.

[bb12] Rangan, V., Perumal, T. E., Sathishkumar, K. & Uppu, R. M. (2006). *Toxicologist* (supplement to *Toxicol. Sci.*) **90**, 242–243. https://www.toxicology.org/pubs/docs/Tox/2006Tox.pdf

[bb13] Salahifar, E., Nematollahi, D., Bayat, M., Mahyari, A. & Rudbari, H. A. (2015). *Org. Lett.* **17**, 4666–4669. 10.1021/acs.orglett.5b01837 26381590

[bb14] Sheldrick, G. M. (2008). *Acta Cryst.* A**64**, 112–122. 10.1107/S0108767307043930 18156677

[bb15] Sheldrick, G. M. (2015). *Acta Cryst.* C**71**, 3–8.

[bb16] Uppu, R. M. & Martin, R. J. (2005). *Toxicologist* (supplement to *Toxicol. Sci.*), **319**. https://www.toxicology.org/pubs/docs/Tox/2005Tox.pdf

[bb17] Uppu, R. M., Sathishkumar, K. & Perumal, T. (2005). Free Radic. Biol. Med. 39 (Suppl. 1) 39, 15. https://www.sciencedirect.com/journal/free-radical-biology-and-medicine/vol/39/suppl/S1

[bb18] Westrip, S. P. (2010). *J. Appl. Cryst.* **43**, 920–925.

